# Living Cell Microarrays: An Overview of Concepts

**DOI:** 10.3390/microarrays5020011

**Published:** 2016-05-26

**Authors:** Rebecca Jonczyk, Tracy Kurth, Antonina Lavrentieva, Johanna-Gabriela Walter, Thomas Scheper, Frank Stahl

**Affiliations:** Institute of Technical Chemistry, Leibniz University of Hannover, Callinstr. 5, Hannover 30167, Germany; rjonczyk@iftc.uni-hannover.de (R.J.); kurth@iftc.uni-hannover.de (T.K.); lavrentieva@iftc.uni-hannover.de (A.L.); walter@iftc.uni-hannover.de (J.-G.W.); scheper@iftc.uni-hannover.de (T.S.)

**Keywords:** living cell microarrays (LCMAs), transfected cell microarrays, affinity-based arrays, cell attachment and stimulation, 3D cell constructs, microfluidic devices, organ-on-a-chip (OOC)

## Abstract

Living cell microarrays are a highly efficient cellular screening system. Due to the low number of cells required per spot, cell microarrays enable the use of primary and stem cells and provide resolution close to the single-cell level. Apart from a variety of conventional static designs, microfluidic microarray systems have also been established. An alternative format is a microarray consisting of three-dimensional cell constructs ranging from cell spheroids to cells encapsulated in hydrogel. These systems provide an *in vivo*-like microenvironment and are preferably used for the investigation of cellular physiology, cytotoxicity, and drug screening. Thus, many different high-tech microarray platforms are currently available. Disadvantages of many systems include their high cost, the requirement of specialized equipment for their manufacture, and the poor comparability of results between different platforms. In this article, we provide an overview of static, microfluidic, and 3D cell microarrays. In addition, we describe a simple method for the printing of living cell microarrays on modified microscope glass slides using standard DNA microarray equipment available in most laboratories. Applications in research and diagnostics are discussed, e.g., the selective and sensitive detection of biomarkers. Finally, we highlight current limitations and the future prospects of living cell microarrays.

## 1. Introduction

**Scheme 1:**
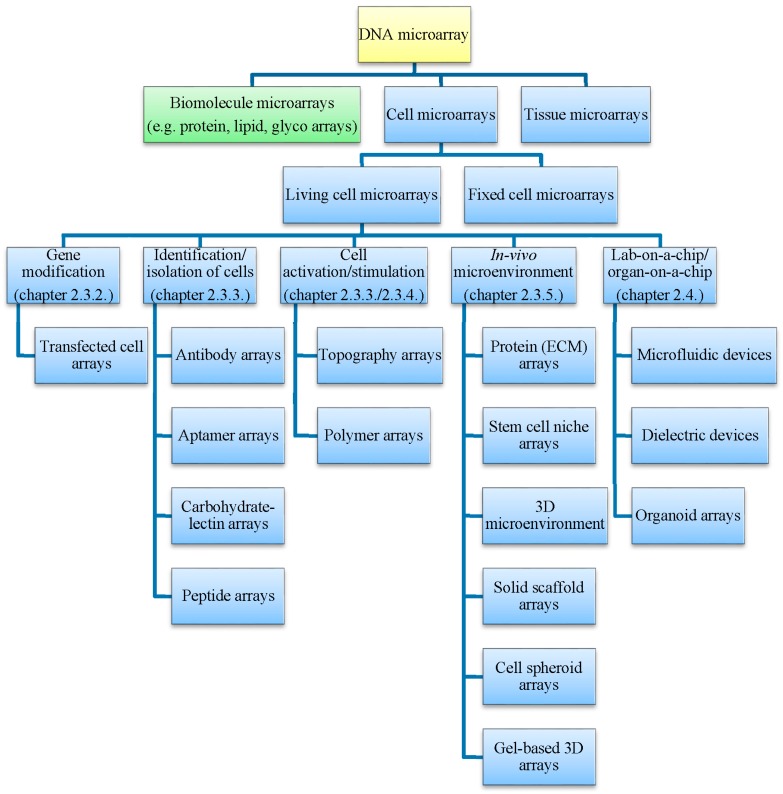
Overview of living cell microarrays presented in this review.

Microarrays became standard tools for gene expression analysis in the 1990s. They combine high-throughput screening and miniaturization on the same platform, and were adapted to many kinds of biomolecules/biological samples like proteins, antibodies, small molecules, cell lysates, fixed tissues, living cells, and organoids. In a microarray, probes are arranged in rows and columns on a solid slide surface in a highly reproducible pattern, in order to allow easy and precise identification. All microarray formats share a common principle: well-defined probes are fixed onto a surface at defined positions, and a mixture of multiple components is applied. Recognition events are detected by fluorescence or by an electrochemical signal. There are sophisticated developments towards micro-electromechanical systems (MEMS) and lab-on-a-chip systems, but for standard microarray experiments performed on modified glass slides, the major equipment consists of a spotter and a scanner. To transfer a standard microarray experiment to living cells, the microarray surface should primarily be suitable for the attachment, proliferation, and differentiation of living cells. Furthermore, the storage of printed living cell microarrays must be optimized. Apart from these challenges, living cell microarrays can be produced, processed, and analyzed with standard microarray equipment. Two- and three-dimensional cell-based microarrays on modified slides are the focus of this paper. Additionally, we discuss recently developed specialized platforms, such as microfluidic devices, as well as lab-on-a-chip/organ-on-a-chip (OOC) applications. An overview about the applications of living cell microarrays (LCMAs) is provided in Scheme 1.

## 2. Literature Review Section

### 2.1. Historical Background

Initially, microarray technology was developed to investigate gene regulation via DNA microarrays [1]. In this format, 10^5^ short oligonucleotides are immobilized on a surface-modified slide at defined locations (spots) to serve as capture molecules (probes). Here, each spot represents one gene. Immobilization, ranging from a few dozen genes up to the immobilization of the whole genome of an organism on a single slide, results in a high-throughput analyzing method. Two different fluorescently labeled DNA- or reverse-transcribed mRNA samples, which are isolated from the control and diseased or experimental tissues or cells, function as targets. These molecules bind and hybridize to their corresponding capture molecules on the microarray. The fluorescence intensity of each spot is quantified and is proportional to the amount of fluorescent targets, which hybridized on the microarray. Therefore, the determined ratio of the different fluorescent DNA-targets represents the relative abundance of DNA or mRNA in the investigated cells [2,3]. DNA microarrays are used in clinical diagnostics for the profiling of gene expression in tissues and cells, the characterization of diseases, and their corresponding genetic risk factors and for the identification of biomarkers for cancer treatment. Furthermore, with the help of these microarrays, gene polymorphisms, as well as the gene expression of markers involved in drug-metabolism or toxicology can be studied [4,5]. To permit accurate probe detection, special fluorescence scanners for glass objective slides were established, which provide high sensitivities and a good resolution of the defined circular spots. Since all samples on the microarray are treated under identical conditions and are incubated with the same amounts of analyzing reagents, the hundreds or thousands of results of one slide are highly comparable to each other. At the same time, only short processing times and low material amounts are required. The density of information is controlled by the number of spots, while the reliability of detection depends on the distance between spots [6].

Protein microarrays were historically established to compensate for the fact that there can be discrepancies between mRNA and protein levels detected in cells at a given time [7,8]. The first protein microarrays used antibodies; later, platforms also employed aptamers (short single-stranded oligonucleotides) as capture molecules on the microarray surface [9,10,11]. In an alternative setup, the target proteins can be directly printed onto the microarray surface, where they are detected by mobile capture molecules [11]. Molecules can be immobilized on the microarray surface by the use of computer-controlled spotters, either via direct contact of a pin to the microarray’s surface or by using a contact-free automated pipette [12,13].

### 2.2. Tissue and Cell Microarrays

To improve the direct phenotyping of cells from tissues, high-throughput tissue microarrays were developed [14] and optimized in the late 1990s [15]. Previously, tissue sections were used for this purpose, which had to be individually examined in a manual manner. This technique requires high amounts of antibodies and reagents, as well as large quantities of limited tissue probes. By the optimization of tissue microarrays (TMAs), the number of examinations performable with one single biopsy probe increased from 100 to 500,000 tests [16]. Thus, TMAs became the method-of-choice for protein and cells staining in tissues or for the validation of cDNA microarray results in experiments seeking the identification of new biomarkers and therapeutic target proteins [16,17]. A variety of analytic methods can be used to analyze the TMAs, e.g., immunohistochemistry (protein detection via enzyme-linked antibodies), immunofluorescence staining (protein detection via fluorescent-labeled antibodies), and fluorescence *in-situ* hybridization (determination of the copy number of genes via fluorescent-labeled DNA-fragments complementary to a gene) [16,18]. The recently published review of Vogel gives an overview of the milestones in TMA preparation from 1986 to 2014 [19].

In general, a hollow needle takes tissue core samples out of a donor tissue block, which is fixated in formalin and embedded in paraffin. The tissue core samples are then placed in an empty acceptor block at defined positions. Further tissue core samples from other donor blocks are transferred to the acceptor block. The acceptor blocks prepared in this manner are cut into sections by a microtome, after which the sections are placed on glass microscope slides [16]. In a single investigation, a variety of up to 10,000 tissue core samples can be simultaneously stained and analyzed under identical conditions. Two or three core samples from the same donor block per TMA ensure representative results of the biopsy probe [20]. Each tissue microarray can be arranged individually, in order to investigate a specific experimental question, resulting in TMAs containing, e.g., tumors of the same type in different stages of the disease [18,21]. In addition to tissue samples, well-defined and standardized controls consisting of native/healthy tissues or cell lines are used to enable a quantitative comparison of microarray experiments between different laboratories and dates [16,18]. The malignant transformation, differentiation, and other cellular processes of adherent cell lines are well-known and thoroughly described in the literature. As a result, adherent cell lines serve as an ideal control in tissue microarrays [22,23,24].

Pure cell microarrays (CMAs) are used for the easy identification of controls for immunostaining (positive or negative control) and for assay optimization by replacing expensive tissue probes. Protein expression profiles of whole cells, the effects of drug treatments, or other stimuli, as well as the effects of gene silencing experiments, were identified in 2005 using this method [25,26,27]. For this purpose, the cells were stimulated, fixed in formalin or paraformaldehyde, and then embedded in paraffin, agar, or low-melting agarose. These cell blocks serve as donor blocks for the creation of microarrays comparable to TMAs [26,27,28,29]. La Spada and coworkers [30] reported a preservation of elongated cell morphology in the prepared CMAs after fixation and scraping of induced pluripotent stem cells (iPSC) differentiated into neuronal lineage. Furthermore, they described an easier detection of the protein markers, as well as better image analysis, and thus a reduction of misinterpretation of the immunofluorescence staining of cell microarrays [30]. Stimulated and fixed cells can be directly transferred to microarrays utilizing a contact nanoprinter [31,32].

The procedure of fixation and embedding in paraffin influences the quality of cells and tissues, as well as the reproducibility of results, depending on the fixation time and antigen recovering protocols. Thus, the *in-situ* analysis of DNA, RNA, or proteins can lead to incorrect results [33,34]. In order to prevent this, frozen cell and tissue microarrays were developed [35,36,37]. Freshly frozen cells and tissues, however, lose their structure, resulting in severe alterations in cell morphology [35].

### 2.3. Living Cell Microarrays

Several research groups have established microarrays of living mammalian or prokaryotic cells over the last few years. In 2001, Ziauddin & Sabatini [38] laid down the basis for living cell microarrays. They printed DNA at defined locations on a microarray. Mammalian adherent cells grew on the printed area and took up this DNA. Thus, spots of localized transfection were created, which led to the rapid discovery of gene functions and the identification of drug targets, as well as gene products [38]. Further developments of the first transfected cell microarrays (presented in Section 2.3.2) led to the dissemination and application of this high-throughput screening platform to several research areas. Angres and the working group of Belkin gave detailed insights into the first steps of the evolution of whole-cell arrays [39,40,41]. In contrast to the working group of Belkin, which specializes in the development of biosensor arrays consisting of genetically tailored microbial cells, our review will focus exclusively on mammalian cells.

All microarrays using living cells instead of purified cellular components are utilized to monitor complex, functional, and vital cellular responses. Living cell microarrays (LCMAs) were applied to characterize cell–cell interactions, cell interactions with their microenvironment, and reactions to applied stimuli, as well as to gain insight into molecular cellular mechanisms [42]. With the help of LCMAs, cells can be easily characterized with regard to their surface molecules. Cell activation caused by external stimuli can be detected in terms of intracellular signaling, inducing transcription as well as translation of genes, cell differentiation and proliferation, or cell death [40,43]. In this manner, toxic, genotoxic or other effects of biologically active molecules can be determined faster. Thus, at the beginning of a drug development process, a huge number of drug candidates and their *in-situ* enzyme-generated metabolites can be screened, resulting in a more efficient development of drugs [43,44,45]. Effects on gene or protein expression can be investigated after the extraction of DNA, RNA, or proteins from the cells [46,47]. However, such a miniaturized format can represent a challenge for the isolation of sufficient amounts of analytes to be studied. The isolation of DNA and RNA from small biomass quantities was successfully performed by Mutiu *et al.* [48]. The nucleic acids can be isolated from the whole cell population or after separation of individual living cells using high-speed cell sorting. Combining flow cytometric analysis and sorting of living cells with transcriptome analysis helps to relate molecular regulation processes within cellular subpopulations with the dynamics of the whole cell population [49].

A non-destructive observation of cell responses in real-time is easily feasible. Therefore, the applications range from basic cell biology studies to sophisticated drug testing procedures. Responses of the LCMAs are visualized in two different ways [43,50,51]: either label-free by electrochemical detection methods, such as surface plasmon resonance (SPR), differential interference contrast (DIC) microscopy, electric cell-substrate impedance sensors (ECIS), and magnetic resonance imaging (MRI), or by optical techniques using fluorescent/bioluminescent probes for specifically staining cellular targets. In the latter techniques, detection is performed by a fluorescence microscope or by a high-resolution fluorescence scanner [43,50,51]. Multi-electrode arrays (MEAs) can be additionally utilized as an electrochemical detection method. Moreover, they are a powerful tool for studying the electrophysiological effects of stimuli to single cells in neuroscience and cardiology [52,53,54]. For more information concerning microbial and mammalian cell biosensors, we would like to refer to a review article dealing with the construction of cell microarrays for biosensing purposes [55]. Furthermore, there are two recently published books focusing on the devices, cells, and applications of whole cell sensing systems [56,57].

**Figure 1:**
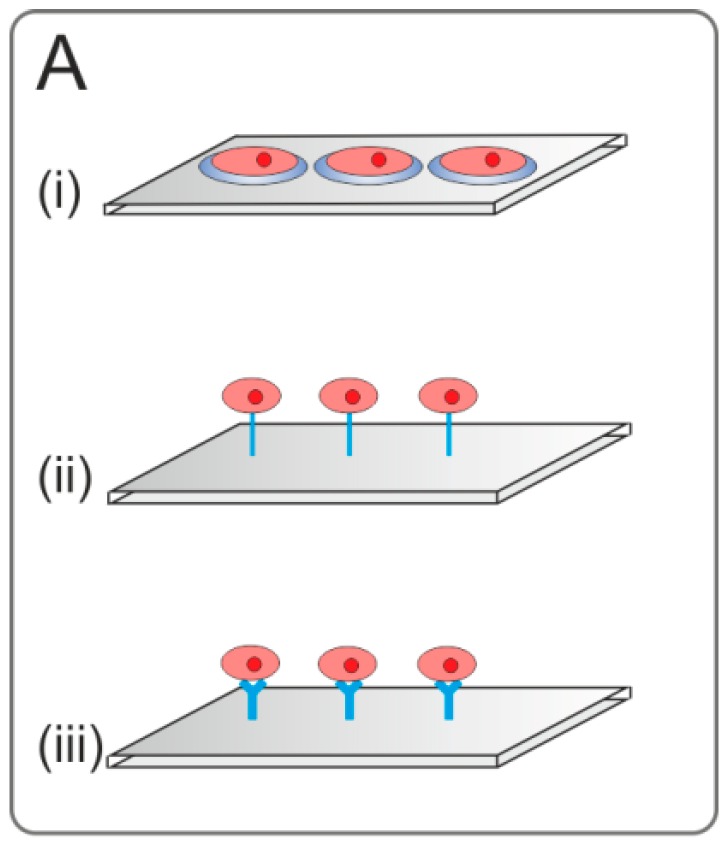

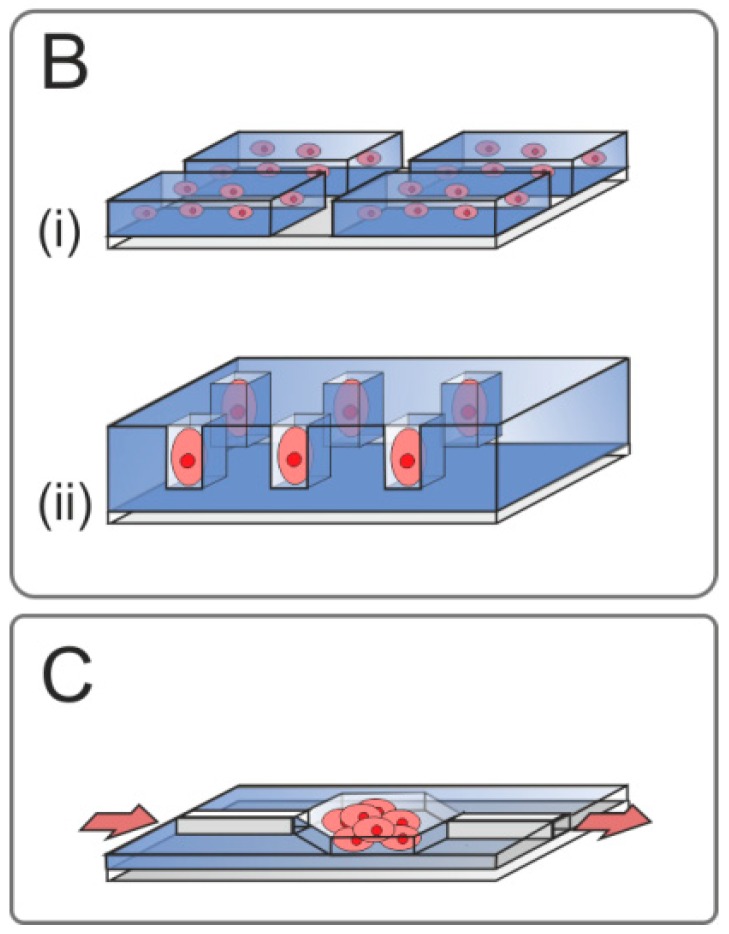
Figurative overview of living cell microarrays (LCMAs) described in this review. (**A**) 2D platforms; (**i**) undirected adsorption of cells on passivated LCMAs with partially unpassivated surface areas; (**ii**) LCMAs coated with biomaterials for enhanced cell attachment and stimulation, inclusively transfected cell microarrays; (**iii**) affinity-based immobilization of cells; (**B**) LCMAs for simulations of *in vivo* microenvironments and cell stimulation; (**i**) 3D cell constructs; (**ii**) microarrays with microstructured surfaces; (**C**) microfluidic devices with organoids.

The preparation of a LCMA requires a stable attachment of cells or cell-binding biomolecules to the microarray surface, as well as preventive measures for avoiding cross-contamination. Microarray surfaces should not interfere with cultivated cells in order to ensure meaningful results of stimulation experiments [42,58]. In addition, the microarray surface has to be chemically and physically stable, preventing cross-reactivity with medium ingredients or changes during the sterilization procedure [59]. The preparation strategies for LCMAs can be divided into two different techniques. In the first method, the cell suspension is positioned at defined locations on the microarray [60], which is further described in Section 2.3.1. In the second method, the microarray is modified to allow cells exposed to the surface to adhere only at defined spots [61,62,63]. Different applications of both techniques are introduced in Section 2.3.3, Section 2.3.4 and Section 2.3.5. Angres [58] introduced these two main techniques for LCMA preparation with the termini “genuine” and “substrate-based cell arrays”. In addition, she subdivided the first type into arrays of dielectrophoretic-positioned and printed cells. The latter category contained different types of microarrayed biomolecules (antibodies, peptides, glycans, proteins, and lipid membranes), as well as transfected cell microarrays. In our review, we will track Angres’ categories roughly, while presenting additional aspects ranging from cell-specific ligands and adsorption to entrapment or encapsulation [58]. New developments, such as three-dimensional (3D) cell constructs are described in Section 2.3.5, which were applied to LCMAs to create *in vivo*-like structures for better and more reliable models. The next evolutionary step of this microarray type was the recent combination of microfluidics and 3D cell constructions to generate lab-on-a-chip platforms. These specialized systems are described in Section 2.4, and a figurative overview of some of these systems is given in Figure 1.

#### 2.3.1. Microarraying of Cells

Cell microarrays are generated by a variety of cell patterning techniques including printing, photolithography, soft-lithography, and dielectric, microfluidic devices [64,65,66]. Here, we will describe cell printing either by contact or contact-free inkjet printing. As contact printing can damage the cells mechanically, this cell-patterning method is commonly used for fixed cells [31]. In contrast, contact-free devices do not directly interact with the cell sample and are thus feasible for the printing of living cells. Cell suspensions have been printed utilizing a contact-free printer in few studies only. Although the shear stress applied to cells in a droplet of liquid during the printing process should be considerable, in general, high cell viabilities have been observed [60,67,68]. In the first reported study, a modified computer printer was used for cell patterning [67]. A variety of cell types, as well as isolated single-cells in a droplet of few picoliters can be printed at defined locations of a single microarray via inkjet technology [69,70]. The application of recently established piezoelectric non-contact nanoprinters provided us with the possibility to transfer very small amounts of living cells (1200 cells and fewer) to microscope slides and to subsequently cultivate and monitor the applied cells online [60]. Here, one cell per droplet (0.4 nL) was printed. We used an incubation chamber to divide the microarray into 16 separate wells, in which the cells grew in clearly defined circular spots in a manner comparable to standard conditions. Since inkjet printing technology offers the possibility of printing cells on different surface topographies, cells can be transferred to slides coated with specific proteins, to topographic structures for enhanced attachment, or to microtiter plates, if preferred [60].

By increasing the viscosity of the printing solution, it is possible to prevent settling and aggregation of cells before and during the printing process. This leads to a standardized and reproducible dosage of cells. The addition of glycerol or sugars is the simplest method to increase viscosity in this case [31]. More complex systems, known as bio-inks, use the cross-linking reaction of alginate and calcium ions or fibrin and thrombin, to rapidly form a gel [50,71]. These bio-inks are not suitable for every cell type. Therefore, Ferris *et al.* [72] recently applied a bio-ink based on gellan gum and surfactants in culture medium to print different cell types. All of these bio-ink systems are suitable for the preparation of multiplex cell microarrays and single-cell microarrays [73,74]. The main advantages are the spatial separation of cell types and the 3D microenvironment provided for the cells. Proteins, such as collagen or fibronectin, are often added for enhanced cell-matrix interaction [75]. Thus, even single cells have contact to the matrix in all three dimensions. Bio-ink systems are also the basis for layer-by-layer printing of 3D structures [50]. Recently, state-of-the-art articles were published describing several methods used in 3D bioprinting technology [76,77].

#### 2.3.2. Transfected Cell Microarrays

In 2001, Ziauddin & Sabatini [38] printed different sets of complementary DNAs, which were cloned in vectors and solved in aqueous gelatin, onto a glass slide. The aim of the experiment was to investigate a variety of cell reactions and changes in cellular behavior as a result of the activation of genes. The DNA spots were dried and treated with a lipid transfection reagent, resulting in a lipid-DNA complex. Afterwards, the microarray was covered with adherently growing HEK293T cells in medium. The oligonucleotides were incorporated into the cells via reverse transfection. The transiently transfected cells on the microarray express the genes and can be fixed, analyzed (by *in-situ* hybridization, immunofluorescence, or autoradiography), and visualized, e.g., by a laser fluorescence scanner or by a fluorescence microscope [38]. One spot on the microarray consisted of 30–500 transfected living cells. Since each spot was 120–250 µm in diameter, a spot density of up to 10,000 spots per standard glass slide was feasible [78]. Total expression levels directly depended on the applied amount of plasmid DNA, whereby only transfectable cells, such as HEK293T cells, could be used. HeLa and A-549 cells compared unfavorably, which was related to poorer transfection efficiencies [38]. By optimization of the transfection protocol for each cell type, the transfection efficiencies were improved, and a variety of cell types have been investigated successfully to date [78]. An alternative composition of the printed gel solution consisted of oligonucleotides, gelatin, and the lipid transfection reagent [79,80,81]. The addition of sucrose enhanced the storage stability of the printed microarrays up to 15 months. Fibronectin or other proteins reduced cross-contamination of transfected cells due to enhanced cell adherence and minimized migration. Furthermore, transfection efficacy was increased [79,80,81]. Thus, even primary cells were successfully transfected in an efficient and nontoxic procedure [80]. Detailed protocols were described by Erfle and coworkers [82]. Since the complete slide surface is covered with a cell carpet, a successful gene transfer into the cells could be easily observed by the additional expression of a reporter gene, e.g., green fluorescent protein [38]. This procedure results in fluorescent cells at the spots of oligonucleotides and non-fluorescent cells attached between the oligonucleotide spots [38,42,83,84,85]. Other research groups used GFP-transfected cells for creating a kind of grid between the non-fluorescent cells used in their studies [38,86]. Co-transfections of more than one gene into the same cell were achievable [38,79,87].

Transfected cell microarrays were suitable for the rapid and systematic high-throughput screening for genes that cause a desired phenotype or encode the products of interest. Ziauddin & Sabatini [38] demonstrated the proof-of-principle by the evaluation of binding specificities of a dopamine antagonist to a cell membrane protein, as well as by the detection of a pharmacologically relevant target of an immunosuppressive drug [38]. Furthermore, screening of transfected cell microarrays resulted in the identification of several proteins, which are involved in apoptosis, cell adhesion, and the kinase-signaling pathway [38,86,87]. Transfected cell microarrays were also utilized to analyze the effects of human herpesvirus-8 on a cellular transcription factor pathway [88]. Recently, this method was used to identify a preselection of highly relevant gene candidates for pancreatic cancer [89]. Unfixed, alive transfected cells served to investigate in-time cellular processes such as protein or organelle dynamics, kinetics, translocations, and redistribution or changes of phenotype over time [90,91].

Besides gene activation and overexpression, the study of gene downregulation and silencing can be performed with LCMAs as well. RNA interference (RNAi), established as a transient or as a stable process, has been used as a post-transcriptional loss-of-function tool since 2003. For a deeper look inside this method, we would like to refer to other reviews and articles [92,93,94]. In brief, synthetic small interfering RNAs (siRNAs) sequence-selectively suppress the genes of interest by base-pairing of the anti-sense nucleic acid to the target mRNA, which initiates its degradation [95]. Similar to cDNA transfected cell microarrays, siRNA was dissolved in aqueous gelatin mixed with the lipid transfection reagent and printed onto glass slides. Likewise, sucrose and fibronectin were added for enhanced transfection efficiency [96,97,98]. Nevertheless, many cell lines, especially primary cells with only a few exceptions, are obstacles to standard transfection methods. Thus, a stable integration and gene suppression tool was needed for RNAi screenings. Here, a viral transduction of short hairpin RNA (shRNA) vectors was implemented [78]. The vectors are taken up by the cell and integrated into the genome. Thereafter, shRNAs are produced in the cell nucleus as two complementary RNA sequences linked by a short loop. A RNAse III enzyme cleaves the shRNA into siRNAs, which then initiate mRNA degradation [93,99].

According to the cDNA cell microarrays described above, gene expression on RNAi cell microarrays was sequence-specific regulated, whereby the expression level was time- and dose-dependent and limited to the discrete spots [97]. The resulting cell microarrays are also known as solid-phase optimized transfection RNAi. An overview of the different high-throughput RNAi screenings in cultured cells can be found in reviews [78,100]. Additionally to reports where RNAi was used as a proof-of-concept to efficiently suppress the expression of reporter genes [97,101] or their fusion proteins [102], the research group of Erfle used transfected cell microarrays for the investigation of a variety of cellular regulation processes including specific endocytosis or secretory pathways [79,82,103]. Co-transfection using unspecific Cy3-labeled DNA oligonucleotides can be used to locate the exact position of transfected cells with only minor effects on uptake and efficacy [79]. In a recent study, researchers used stable fluorescent HeLa cells for the automatic and time-resolved investigation of cellular division, proliferation, survival, and migration via changes in phenotype after incorporation of RNAi [91].

Rantala *et al.* [81] and Fengler and coworkers [104] developed a cell spot microarray method, which supports the preparation of transfection cell microarrays and the analysis of RNAi screenings. Similar to Erfle and coworkers [96], they added fibronectin or a protein mixture for an enhanced, faster cell attachment at the oligonucleotide spots. Rantala *et al.* [81] additionally applied the transfection solution to a plate with hydrophobic polystyrene. After cell seeding and an adherence time of 5–20 min, a washing step was introduced into the microarray preparation procedure to remove all non-adhered cells between the spots. In this way, spots of transfected cells are separated from each other, which supports the automated analysis better compared to microarrays of a cell carpet [81,104]. Where data analysis are facilitated in this way, the differently treated cell groups on one microarray are not separated from each other, which may cause stimulation across the cell spots induced by secreted factors. This first step in the prevention of cross-contamination of different transfected and wildtype cells, however, is not usable for the investigation of different cell types on one slide. Further developments involving the increase of the variety of applied cell types are described in the following chapters.

#### 2.3.3. Affinity-Based Immobilization

The construction of a LCMA containing different cell types demands for stable immobilization of the cells. Therefore, ligands targeting cells with high affinity and specificity are helpful. The affinity-based immobilization of cells is an effective way to specifically capture cells that differ in type, stage of differentiation, or development during malignant transformation. Capturing specific cells provides important information for analyzing cellular processes. Immobilizing different capture molecules to microarrays allows the identification of cell types based on the expression of different cell surface molecules [43]. Another application of cell microarrays is the capture of specific cell types out of a complex cell mixture [105]. Cell-specific capture reagents include antibodies, proteins, aptamers, peptides, and small molecules [105].

Mammalian cells possess a complex array of glycans on the cell surface. The entity of glycans expressed in a cell is called the glycome. It comprises glycolipids, glycoproteins, and proteoglycans. It has been assumed that the expressed set of glycans differs between different cell types and different stages of cell development and differentiation [106]. Furthermore, malignant transformation alters glycosylation [106]. Lectins and glycans can be spotted onto microarrays for cell carbohydrate or lectin profiling [43]. A method for glycan profiling of living mammalian cells using lectin microarrays was established by Tateno *et al.* in 2007 [106]. Most of the interactions between carbohydrates and lectins on the cell surface are multivalent. This multivalency, as well as spotting lectins or carbohydrates in high concentrations, promotes cell binding even further. This simple method allows a rapid profiling of the cell surface glycome and is important for the understanding of glycome changes in cellular processes including cell-to-cell communication and immune response modulation [43,106]. Furthermore, the identification, analysis, characterization, and capture of different cell types with lectin microarrays is a useful tool in various fields, such as medicine and biology [105]. It is estimated that mammalian cells possess about 500 unique glycan structures and that the cell surface displays about 100 different lectins [43]. The microarray described by Tateno *et al.* [106] consisted of 43 lectins with distinct binding specificities, which were spotted separately and covalently bound to an epoxy array. The slide was incubated with Chinese Hamster Ovary (CHO) cells and their glycosylation-defective mutants (Lec1, Lec2, and Lec8), K562 cells after and before differentiation, and the splenocytes of wildtype and β1-3-*N*-acetylglucosaminyltransferase II knockout mice. The cells were labeled with Cell Tracker Orange and detected with an evanescent-field fluorescence scanner in the liquid phase. The experiments showed that lectin microarrays require a relatively low amount of cells, the glycome can be characterized in an intact state, experiments can be carried out in a high-throughput and rapid manner, and cells remain viable, which is useful for functional analysis [106].

Another method allowing the rapid and efficient analysis of cells is an immunoaffinity-based microarray. Milgram *et al.* [107] developed an antibody microarray for label-free cell-based applications in 2011. The study was focused on the microarray fabrication for SPR analysis of cells or cellular activity. Therefore, immunoglobulin G (IgG) antibodies, anti-CD90 (Cluster of Differentiation 90) against T-lymphocytes (LS102.9), and anti-I-A against B lymphocytes (3A9) were separately conjugated to *N*-Hydroxysuccinimide (NHS) pyrrole. The solutions of coupled antibodies were diluted in spotting buffer containing the free pyrrole. The antibody immobilization on the gold-covered glass slide occurred through electropolymerization of the pyrrol coupled to the antibodies with free pyrrole. Then, the slide was incubated with a mixture of B- and T-lymphocytes. With the help of a fluorescently labeled antibody (phycoerythrin anti-CD19), which specifically binds B-lymphocytes, the specific capturing of cells could be confirmed through optical and fluorescence microscopy. The polypyrrole-based chemistry proved to be an efficient and robust method immobilizing antibodies on biochips for label-free cellular analysis [107].

Apart from antibodies, alternative binders can be used to capture cells on LCMAs. Aptamers are single-stranded oligonucleotides, which bind their target molecules with high affinity and specificity. In comparison to antibodies, aptamers display higher long-term stability [11]. Another advantage is the low toxicity of aptamers [108]. Aptamers can be easily synthesized and modified for direct immobilization on microarrays [11]. For example, Song *et al.* [109] showed that circulating tumor cells (CTC) expressing the Epithelial Cell Adhesion Molecule (EpCAM) can be detected and captured by the DNA aptamer SYL3C, which they generated for this purpose. Anti-EpCAM antibody-functionalized surfaces already exist in microfluidic devices, but the use of the antibodies is restricted, because of their size and instability. Therefore, the 3′ terminal biotinylated aptamer SYL3C was immobilized through biotin-streptavidin interactions. To avoid interactions between the immobilized ligand and the surface, a polyethylene glycol (PEG) linker was incorporated to the aptamer between the nucleotides and the biotin moiety. The EpCAM-positive cell line Kato III and the EpCAM-negative cell line Ramos were used for the experiments. Aptamer SYL3C was shown to possess the potential for CTC enrichment and could be used on microarrays [109].

In another study performed by Chen *et al.* [110], an aptamer targeting Ramos cells was used to isolate and analyze single tumor cells with microwell arrays on a microfluidic device. The microwell array was coated with avidin to bind the biotinylated aptamers. The captured cells were observed under the microscope. Enzyme kinetics were analyzed using cell-permeable dye to monitor the intramolecular fluorescence of the cells with a fluorescence microscope. The aptamers-coated microwell array on the microfluidic device was shown to be capable of separation between specific tumor cells and could be of use for clinical samples, e.g., blood samples. In the future, personalized medicine could be established through specific tumor cell (isolated from the certain patient) enzyme kinetics in high-throughput assays. This novel method offers new opportunities in clinical applications [110].

In another aptamer-based study, anti-prostate-specific membrane antigen (PSMA) aptamers were immobilized on a microchip and fabricated into a high-throughput micro-sampling unit by Dharmasiri *et al.* [111] to capture rare circulating prostate tumor cells. Therefore, the poly(methyl methacrylate) surface of the microchip was activated using UV light. The generated carboxylic acid was then converted into a succinimidyl ester derivate via 1-Ethyl-3-(3-dimethylaminopropyl)carbodiimide (EDC) coupling, which then reacted with amino-modified aptamers. Anti-PSMA aptamers target LNCaP cells (prostate cancer cell line). LNCaP cells were counted, fluorescently labeled, and put into whole blood with CTC concentrations of 10 cells per mL. The cells could be successfully released after selecting and capturing via enzymatic digestion of the extracellular membrane (ECM) domain of PSMA using trypsin. The anti-PSMA aptamer was found to bind LNCaP specifically and is of use for capturing cells out of complex mixtures with high-throughput micro-sampling devices [111].

Small-molecule and peptide microarrays were used for screening living mammalian cells by Lee *et al.* [112]. Ligands were conjugated to the surface via polyethylene glycol—*N*-Hydroxysuccinimide (PEG-NHS). The PEG linker presents the ligand to its target with a distance to the microarray surface. All spotted ligands contained a primary amino group. The peptide cRGDyk is known to bind the integrin αvβ3 receptor. After binding the peptide cRGDyk to the surface, receptor positive and negative M21 cells were labeled with fluorescent dyes differently and incubated on the microarray to prove the specific binding of the peptide. Other specific ligands used by Lee *et al.* [112] to optimize the assay for high-throughput applications are known to be specific for the PSMA receptor, e.g., β-AG and Glycophosphatidylinositol. Another ligand specifically binding melanocortin-1 receptors is the α-melanocyte stimulating hormone, which was also used by Lee *et al.* [112].

Falsey *et al.* [113] prepared chemical microscope slides for the covalent immobilization of small molecules and peptides through site-specific oxime bond or thiazolidine ring ligation reaction. Glass slides were derivatized with (3-Aminopropyl)triethoxysilane (APTES). Two different ways to convert the amino slides to glyoxylyl derivates were used. One of them was to couple fluorenylmethoxycarbonyl serine followed by deprotection and oxidation. The second was to couple protected glyoxylic acid to the slide and deprotect it afterwards with hydrochloric acid. Biotin and the peptides were modified before spotting. The carboxyl terminus was coupled to a succinimic derivate as linker and an amino-oxy group or 1, 2-amino-thiol group. Stearic acids were added separately during the coupling step with glyoxylic acid to avoid unspecific binding. The peptide wGeyidvk was found to specifically bind to the surface idiotype of WEHI-231 cells (murine lymphoma). For the cell adhesion assay, Falsey *et al.* have immobilized the peptide wGeyidvk on the microarray. The labeled target cells were incubated on the microarray and were shown to specifically bind to the peptide. PEG_5000_—NHNH_2_ was essential to block the slide and avoid non-specific binding of cells and improve the background for cell adhesion assays [113].

Folic acid, another small molecule, was recently used to modify Montmorillonite clay (FA‑Mont) [114]. FA-Mont was tested as a cell culture material to provide cell adhesion of folate-positive cells on the clay surface. Cultivating folate-receptor-rich HeLa and folate-receptor-poor A-549 cells in 96-well plates coated with FA-Mont proved FA‑Mont’s ability to discriminate between the two cell lines. The cell adhesion was detected with the help of fluorescence and scanning electron microscopy. Potentially, this method could be applied for cell-on-chip studies by modifying the surface using different ligands, since clays are easy to combine with different functional organic groups. Furthermore, they possess low toxicity and are chemically stable [114].

The aim of affinity immobilization is not only enhanced cell attachment, but also the ability of bioactive molecules or proper combinations of them to activate cellular processes, such as phosphorylation, cell differentiation, the production of proteins, and apoptosis [42,63,115]. Therefore, gentle immobilization of the molecules is crucial, as molecules have to display bioactivity afterwards. Denaturation and conformational changes, as well as misorientation, lead to inefficient binding of cellular receptors and to decreased bioactivity. Thus, covalent binding and self-assembly of molecules to microarray surfaces are often used for long-term studies of cells [55]. The influence of lectin and carbohydrates on cell–cell interactions, communication, and immune response was shown by the specific attachment of T-cells to their corresponding sugar residues on a microarray with a variety of carbohydrates [116]. Immobilized peptides on microarrays likewise cause a response when cells bind to and incorporate these molecules. Here, tyrosine phosphorylation was induced in certain cell types [113]. Simultaneous immobilization of bioactive molecules and antibodies yielded the opportunity to induce protein secretion and detection of secreted proteins by the immobilized antibodies in a single experiment. As the volume of medium contained on a microarray is very small, the detection limit of antibody-protein binding is easily reached under standard conditions. A more detailed summary of the detection of cell activation on microarrays has been given elsewhere [43,51].

The examples summarized in this section demonstrate the diversity of ligands for specific cell capture on biochip surfaces, as well as the broad applicability of these ligands in the construction of cell microarrays. While most studies are rather on the proof-of-concept stage, other studies have already allowed the specific identification, attachment, and isolation of cells from complex biological samples. This specific enrichment of desired cell types on the microarray surface represents a major advantage of affinity‑based cell capture: clinical samples can be directly used for the LCMA fabrication with no need for the prior isolation of the cells of interest. Thus, it can be assumed that affinity-based cell capture will continue to be further developed and will contribute to the construction of future LCMAs.

#### 2.3.4. Adsorption and Entrapment

Instead of the printing of cell suspensions, cells can be applied to the complete microarray surface, which was modified previously. This is the common way to prepare living cell microarrays. These microarrays usually possess a glass or silicon surface that is modified via nanoprinter with several spots of cell-attachment-promoting biological molecules. Besides the aforementioned affinity-based immobilization of cells utilizing antibodies, aptamers, as well as specific proteins or peptides, further biomaterials such as polymers were printed to microarray surfaces to control cell attachment [42,113,116,117,118,119]. The binding of cells to these biomolecules is enhanced, whereas the remaining surface is structured or passivated, making it unattractive for cell attachment. In this way, it is ensured that cell groups are immobilized and separated from each other [61,62,63]. In the following Section, these methods are described in more detail.

##### Structured Surfaces

Cells are generally able to adsorb nonspecifically on many different kinds of solid substrates, while their viability and behavior may be affected either positively or negatively. This is exploited to mimic *in vivo* microenvironments or to prevent cross-contaminations and to separate cell spots from each other in an appropriate way. For these purposes, several methods were developed. The simplest method was already mentioned in Section 2.3.2, a micropatterned surface of cell-repellent and cell-adhesive regions. Here, a cell-adhesion-permitting transfection mixture was spotted onto a cell-repulsive hydrophobic polystyrene surface [81]. In this style, cell-repulsive coatings for surface passivation like PEG can be used for the passivation of surfaces between spots of cell attachment-enhancing proteins [63]. Moreover, elastomeric materials were used to form microwells utilizing micromolding-soft-lithography. Surface patterning via photolithography, soft-lithography via microcontact printing, and stencil-assisted techniques have been summarized elsewhere [120,121]. A silicone stamp is dipped into a solution of PEG-diacrylate on a treated glass slide. This solution was polymerized utilizing UV-radiation, resulting in the formation of a microwell-microarray after removal of the stamp. Cells were seeded and cultivated in these wells [122,123]. Additional hydrogels, such as hyaluronic acid or agarose, are also used for the formation of microwell-microarrays via soft-lithography [124]. An overview of strategies for the preparation of multiplexed LCMAs with separated cell groups of different origin was given in a recently published review [73]. A combination of surface chemistry, soft-lithography, and centrifugation was used to pattern different types of cells on a surface in a fast and controlled way [125]. 1-Hexadecanethiol was printed onto gold surfaces by microcontact printing to create hydrophobic areas. The remaining surface was passivated by ethylene glycol before fibronectin was adsorbed to the hydrophobic areas. After preparation, the treated surfaces were placed in centrifugation tubes. A minimal volume of cell suspension was added, and the tube was centrifuged at 2000 rpm for 1 min to force the cells to adhere. To immobilize co-cultures, a mask positioned at defined regions prevented the attachment of the first cell type, so that the second cell type could be patterned after removing the mask [125].

The working group of Kataoka [126,127,128] established a cell microarray chip for the detection and analysis of mixtures of several cell types via a confocal microarray laser scanner. Their chip consists of 20,944 microchambers (105 μm width, 50 μm depth and 300 μm distance) made of polystyrene. They were able to analyze antigen-specific B-cells, to detect malaria-infected erythrocytes within healthy erythrocytes, and to detect circulating carcinoma cells within a probe of leukocytes in a very specific and sensitive manner, while the tests themselves took less than 1 h [126,127,128]. Reymann *et al.* [129] applied cell arrays with 9216 microwells for reverse transfection studies and separated the cells physically in cavities of a titanium-coated glass slide. Thus, they prevented cross-contaminations and exploited the high-throughput advantages of transfected cell microarrays in a single system [129].

When the heterogeneity of cell populations or the individual cellular responses to stimuli are studied, single cells have to be isolated from each other and analyzed individually. Since several sizes of the stamp utilized for microcontact printing are often available, a small size in the range of a single cell can be used for the generation of single-cell microarrays. In another method, a highly defined hydrogel grid was applied, which was produced by dipping a nylon tissue into alginate solution, followed by a subsequent air- and freeze-drying on the microarray slide [130].

Cell behavior and cell fate of several cell types, such as stem cells or progenitor cells, are guided *in vivo* by the biomolecules of the cellular microenvironment, as well as by topographical cues [131]. The influence of a variety of geometries and sizes of surface structures were studied under the same conditions on one single topography array by Moe *et al.* [132]. The authors described an increased influence of these features on the neural and glial differentiation of primary murine neural progenitor cells, while they distinguished between anisotropic and isotropic topographies [132].

##### Biomaterials for Untargeted Cell Attachment and Stimulation

Besides the targeted attachment of a specific cell type described in Section 2.3.3, the general enhancement of cell attachment itself is also focus of intense research. Several biomaterials, such as polymers, are under investigation for this purpose. An overview of common surface modification strategies is given in [133]. Different cell types (cell lines and embryonic stem cells) were cultivated on microarrays with spots of several polymers to investigate the cell adhesion properties of these polymers, resulting in new coatings for cell culture applications [59,134]. In this way, new synthetic biomaterials for tissue engineering applications were identified [117,134]. In addition to polymers, an agarose coating can be used to prevent unspecific cell attachment. As aforementioned, cell-repulsive characteristics were also found for a surface that is densely coated with PEG [122,135]. On top of this coating, polyurethane or polymers were printed to enhance cell attachment at these defined positions [59]. Anderson and coworkers demonstrated that their polymers support stem cell growth and proliferation in different levels and that only certain polymers were able to promote cell spreading and differentiation [134].

#### 2.3.5. Simulation of *In Vivo* Microenvironment

Several approaches for microarray platforms are currently under development in order to create a physiologically relevant microenvironment. If cell reaction upon stimuli should be obtained, cells have to be provided with passive (stiffness, geometrical clues) and active (biological and chemical signals, cell–cell contacts) microenvironment conditions. For example, the *in vivo* microenvironment of cancer cells is composed of different types of neighboring cells, various chemical gradients, low oxygen concentration, high metabolite concentration, and a mechanical surrounding, resulting in a much more complex system than the one available in a simple monolayer of spotted cancer cells. Moreover, primary cells are sensitive to outer stimuli and change their physiology in simple monolayer cultures in comparison to a true *in vivo* situation. There are two major types of cell-based microarrays, which aim to mimic the *in vivo* microenvironment: (1) cell niche microarrays; (2) 3D cell culture microarrays.

##### Stem Cell Niche

The cell niche is a specific *in vivo* microenvironment to which cells are exposed depending on their location in the body [136,137]. In general, the cell niche is a combination of different physical, chemical, and biological factors. Physical factors include mechanical stiffness of substrate and pressure, chemical factors comprise a distribution of gases, pH values, and nutrients, while biological factors include the interplay of signaling molecules and cell–cell contacts. There are two extensively studied cell niches: the stem cell (SC) niche and the cancer stem cell (CSC) niche. For both, SCs and CSCs, oxygen tension plays an important role since in developing tumors, as well as at injury sites, oxygen tensions are much lower than ambient oxygen concentrations [136,137]. Stem cell niche plays an important role in the understanding of cell biology for regenerative medicine and provides an optimal *in vitro* microenvironment that reflects the *in vivo* situation. In order to simulate the stem cell niche, embryonic stem cells (ESCs) are often cultivated *in vitro* on various feeder cells: mouse embryonic fibroblasts, human fetal muscle and skin cells, adult skin and marrow cells, and foreskin fibroblasts [138]. Induced pluripotent stem cells (iPSCs) are also often cultured with the help of feeder cells. Although co-cultivation with feeder cells can provide cells with complex cytokines and signal molecules, this technique has serious limitations: presence of potential pathogens, risks of cross-contamination, as well as difficulties to distinguish between stem cells and feeder cells in response to external stimuli.

The biochemical and physiochemical microenvironment can be simulated *in vitro* by the usage of ECM proteins and materials with variable mechanical properties. Therefore, several ECM arrays, biomaterial arrays, stiffness arrays, and topography arrays, as well as combinations of these were developed to simulate the complex stem cell niche *in vitro* [131,139,140]. Cellular transmembrane integrin receptors bind to ECM proteins, e.g., collagen and fibronectin. Thus, these molecules as well as polylysine or integrin-binding peptides like RGD (Arg–Gly–Asp, the cell adhesion promoting sequence of fibronectin) are commonly used substrates to control cell attachment. Since the ECM is important for cell adhesion and communication, the effects on cell behavior are well-investigated. Rasi Ghaemi *et al.* [63] investigated the cell adhesion qualities of several ECM proteins using mesenchymal stem cells (MSCs). The authors printed these proteins onto epoxy-silane-coated slides and passivated the residual surface with covalently bound bisamin-PEG. Collagen I was found to be most qualified for cell adhesion and growth, and was also used as surface modification for stem cells undergoing osteogenic differentiation [63]. Likewise, the adhesion profiles of several cell lines were investigated on ECM arrays [141]. Removable microfluidic channels, which are orthogonally aligned to fibronectin-coated gold strips, can be utilized for the generation of replicates of small isolated groups of different cell types on one single microarray [142]. To understand and mimic the microenvironment of cells *in vivo*, defined mixtures of ECM proteins and growth factors are printed onto the microarrays [115]. Microarrays modified with several combinations of ECM proteins and further factors (e.g., matrix stiffness) were used to screen for adhesion profiles of metastatic cell lines and primary tumor-derived cells. Thereby, the interactions between specific integrins of metastatic cells, and the ECM were shown [143]. Specific combinations of ECM proteins affected the differentiation of ESCs and the liver-specific function of hepatocytes [115]. Gobaa and colleagues developed an artificial niche microarray, where stem cell fate could be studied simultaneously under various conditions [140]. In this study, different types of cells (adherent and non-adherent) were spotted on a hydrogel-based microarray platform, coated with different types of proteins and exposed to different stiffnesses (shear moduli in the range of 1–50 kPa) in order to mimic diverse *in vivo* niches/conditions. The authors could reveal the influence of the mechanical environment on stem cell differentiation in terms of specific protein and mRNA expression.

Appropriate simulation of the CSC niche can help to study the mechanisms of tumor development and find suitable treatment strategies. Indeed, most anti-cancer drugs, which were shown to be effective in *in vitro* experiments, demonstrated poor performance in subsequent animal studies and clinical trials. Although many studies have already been performed on the CSC niche and the influence of various parameters on cancer cell biology has been investigated, there are no reports on the establishment of CSC niche microarrays yet.

##### Three-Dimensional Cell Constructs

An efficient separation of cells on the same microarray is possible after encapsulation of the cells. Apart from separation, this method possesses another advantage, since cell growth conditions play a fundamental role in the cellular response to external stimuli [144]. Growth, metabolism, morphology, and organization of adherent cells in *in vitro* cultures differ greatly compared to the processes *in vivo*. *In vivo* cells do not grow in 2D monolayers, but form 3D structures, which can be imitated *in vitro*. Several 3D *in vitro* cell culture platforms have been developed over the last several years in order to improve the physiological relevance of the experimental results and fill the gap between 2D monolayer cell cultures and animal models [75,145]. Organoid cell cultures were first introduced in the early 1970s by radiobiologists in cancer research [146,147]. Being cultivated in cell spheroids, various tumor cell lines demonstrated enhanced resistance to chemotherapy and radiotherapy in terms of decreased apoptosis and increased clonogenecity [148,149]. Numerous studies on various cell lines, including primary cells, revealed significant changes in gene expression profiles for proliferation, angiogenesis, migration, invasion, or chemosensitivity of 3D relative to 2D cell culture conditions [144,150,151,152,153]. A comparison of epithelial cells growing in monolayers or in 3D constructs shows strong distinctions in gene and receptor expression, proliferation, and cell communication, as well as in their properties of differentiation [154,155,156]. Cells, which were grown in 2D culture, recover many of their lost characteristics after implanting them into an *in vivo*-like environment [157]. Due to this, microarrays promoting cell growth in 3D networks became more important in the last few years. There are three major 3D cell culture techniques that are routinely used today: (1) cells grown on a porous (anorganic or protein-based) matrix/scaffold; (2) cell spheroids (organoids); and (3) hydrogel-based cultures [158,159,160]. The choice of the 3D cell culture model is dependent on the intended application—if the aim is to study migration/adhesion in a 3D microenvironment, hydrogel constructs are the best choice. In contrast, for a simulation of a tumor-like environment, organoids are usually used. The main advantage of hydrogels is their transparency, enabling the online monitoring of cell behavior through the entire 3D construct. Several working groups extend traditional 2D living cell microarrays to the physiologically more relevant and more complex 3D systems [161].

Solid Scaffold Microarrays. Solid scaffolds can be created from a variety of materials. It is still discussed if such porous scaffolds provide true or pseudo 3D growth conditions since cells grow attached on the surface of the scaffold and, in general, it is a geometrically more complex structure than a cell culture flask bottom. Ock & Li presented 3D tissue model microarrays fabricated from biodegradable polylactic acid (PLA) [162]. PLA scaffolds were prepared with the help of the laser foaming technique and brain glioblastom T98G cells were seeded and cultivated over different periods of times (up to 120 h). Cells grown in 3D scaffolds demonstrated higher viability and aggregation, and they showed clusters of multiple cells. Moreover, cell–cell connections as well as the formation of microvilli and fibers were observed on 3D cultivated cells.

Cell Spheroid Microarrays. Spontaneous cell–cell aggregation and creation of *in vitro* organoids represents a simple 3D cell culture model. Previously described PEG-microwells, which are formed by soft-lithography, can be used for the formation of 3D cell constructs. Cell repulsion is highest when PEG coating is performed on the complete microarray surface, and the microwells are formed by the hollows in this coating. Seeded cells cannot attach, resulting in cell aggregates, e.g., embryonic bodies of stem cells [123,163]. Cell spheroids of a certain size can be immobilized on the substrate in order to create 3D organoid microarrays. Wang and colleagues established such a microarray system, where they plotted MSC-spheroids on a microdomain patterned template on glass substrates [164]. Cells cultivated in this system were successfully differentiated towards adipocytes. Moreover, the differentiation efficiency in 3D structures was higher than in 2D monolayer controls. Consequently, 3D cultivation conditions are preferable for MSCs and microarray readouts will provide more relevant information about cell response to the stimuli. An alternative 3D spheroid-based approach (gel-free 3D microfluidic cell culture system) was developed by Ong *et al.* [165]. To create cell aggregates under dynamic cultivation conditions, the authors used the inter-cellular polymeric linker polyethyleneimine–hydrazide. Cell lines (A549 and C3A cells) and MSCs aggregates were immobilized in microchannels. Immobilized cells retained their proliferation and differentiation capacity and could be cultivated under dynamic conditions.

Gel-based 3D Microarrays. In comparison to other 3D cell culture platforms, hydrogel-based culture is a relatively new and rapidly developing technique, which allows combination of organic, anorganic, and biological molecules in order to provide cells with an optimal microenvironment. The group of Dordick reported the establishment of 3D microarray platform based on MCF7 and Hep3B cells encapsulated in 60 nL alginate gel spots on modified glass slides for high-throughput toxicity screening [45]. In this study, the authors printed solutions of a cell-gel mixture to microarrays utilizing a non-contact nanoprinter. To polymerize the alginate solution, a mixture of barium chloride (BaCl_2_) and poly-l-lysine (PLL) was printed onto the microarray. The droplets of the cell-gel solution were then spotted over the same positions of previously printed BaCl_2_/PLL. PLL supports the attachment of alginate to the microarray surface, and BaCl_2_ causes the polymerization of the alginate. In a later study, the same working group encapsulated mouse embryonic stem cells (ESCs) in alginate on a microarray [166]. Using this microarray, ESCs could be expanded and differentiated in small format, and the influence of model signaling molecules (tretionin and FGF-4) on these processes was studied in dual slide configuration. The same working group established a 3D microarray for neural stem cell differentiation and toxicology [167] as well as for drug testing [168]. Neural stem cells cultivated in this microarray system could be expanded, differentiated, and used for cytotoxicity testing (dose-response curves) of neurotoxicants (retinoic acid, dexamethasone, and cadmium chloride). Moreover, on-chip in-cell immunofluorescence assay was performed using this microarray, which was comparable to conventional 2D assays. Pathel *et al.* [169] presented bioadhesive maleimide functionalized PEG hydrogel-based microarrays, where adherent and non-adherent cells can be encapsulated. The used microgels supported the spreading and cell growth of HeLa and TF-1a cell lines and could be used for high-throughput drug screening. In addition, the variation in microgel composition provided control of the stiffness for an investigation of the influence of the microenvironment on cell survival and function. Another working group used functionalized PEG hydrogel-based cell microarrays for the cultivation and study of epithelial ovarian carcinoma cell aggregates [47]. Here, the combination of two 3D culturing approaches (termed spheroids in hydrogels) was developed to study cell aggregation and to screen anti-cancer therapeutics. PEG-hydrogels were also used by Ranga and colleagues to create a 3D microarray system for the screening of different environments for mouse ESC cultivation [170]. The authors used an automatic liquid handling robot with nanopipetter head and studied the combination of five 3D microenvironmental factors: mechanical properties, proteolytic degradability, ECM proteins, cell–cell interaction, and the presence of soluble factors. Axelrod and colleagues entrapped single fibroblast cells surrounded by bacterial aggregates in hydrogels on a microarray slide [171]. This microarray system represents a co-culture approach with 3D microenvironment. Fibroblasts, hepatocytes, and macrophages encapsulated in PEG hydrogels, modified with RGD peptides, and immobilized on glass slides were reported by Koh *et al.* [172]. In this work, microgels with all three types of cells were immobilized on one slide in order to create a multiphenotype microarray. Suspensions consisting of cells and PEG were added into a network of microchannels on top of the slide, leading to a separation of different cell types without using a nanoprinter. Ozawa *et al.* [173] used electrodeposition for the fabrication of an alginate gel microwell array, where they cultured ESCs and HepG2 cells to construct spheroids. Moreover, they could create a co-culture system where mouse fibroblast cells were entrapped in alginate layers, and spheroids of another cell type were cultured above. This cultivation approach can also be used as an ESCs niche without the risk of cross-contamination.

Although 3D cell microarrays possess numerous advantages in terms of their biological and physiological complexity, they do bring about challenges in terms of appropriate analytics (like all 3D cell culture systems). The estimation of cell viability, as well as specific staining of the complex structure of live 3D constructs, is still problematic. Moreover, issues of image acquisition, image analysis, and quantification represent additional challenges [174]. Concerning imaging, a sequence of images at a different focus depth (*z*-stack) is required to reveal the full complexity of the 3D organoids. Another approach for 3D microarray imaging is Raman micro-spectroscopy, but this needs further development. In the case of indirect cell viability assays, longer incubation times and often full cell lysis are required. Furthermore, cell assays, which rely on fluorescent and colorimetric assays (e.g., calcium flux), cannot be measured properly with existing methods [174].

### 2.4. Specialized Microarray Systems

Although the application of 3D tissues helps to approach physiological conditions, 3D cell cultivation is still far away from reflecting processes present in the human body. Due to static cultivation conditions, active transport processes are neglected and interaction of different cell types cannot be simulated adequately. To overcome these limitations, more specialized cell microarray systems have been developed. They allow the study of cell biology, including effects based on cell–cell interactions and fluid dynamics by utilizing microfluidic chip systems.

#### 2.4.1. Microfluidic Systems

While conventional multiwell plates are easy to use and are currently the gold standard in cell cultivation and investigation, the true composition of the medium is changing from medium exchange to medium exchange. Due to this, a controlled culture environment and a regulation of nutrient and metabolite concentration is not truly feasible [134,175]. Moreover, static multiwell plate systems can neither reflect the active transport of substances (nutrients, drugs, metabolites, *etc.*) present in the human body, e.g., by the blood circulation, nor imitate the interactions of different cells, tissues, and organs. To enable controlled cultivation conditions, perfusion reactors can be utilized, but they require large volumes of medium and supplements, resulting in expensive and impractical investigations. As a consequence, microfluidic cell culture systems using medium perfusion were developed to provide a controlled culture and differentiation environment [134,175]. Microfluidics allow the precise handling of μL volumes using μm channels in which the fluid flow can be controlled by miniaturized pumps and valves. Originally, microfluidics was introduced for lab-on-a-chip and micro-total analysis systems [176]. Manifold biological assays have already been successfully transferred to microfluidic devices including PCR [177] and protein separation and analysis [178].

For cell culture applications, most microfluidic devices are constructed of polydimethylsiloxane, thereby exploiting the air permeability, plasticity, and biocompatibility of this material [178,179]. In most cases, microfluidic cell culture chips contain at least two cell types at separate positions. The different cell types can, e.g., be cultivated in different chambers of the chip. To allow cell–cell interaction, microgaps can be introduced. These can support communication and migration of cells. For example, Businaro *et al.* [180] have utilized such a system to investigate the cross talk between cancer and immune cells. B16 melanoma cells and immune cells were seeded in different chambers of a microfluidic chip. A microgap interconnected both culture chambers and allowed the observation of immune cell migration towards the cancer cells. Another possibility to separate distinct cultivation chambers is the integration of porous membranes with different cell types seeded on both sides of the membrane [181]. While the membrane represents a physical barrier for the cells, thereby preventing mixing of different cell types, biomolecules like proteins and metabolites can pass through the pores, facilitating the indirect communication of the cells.

Based on the experimental needs, microfluidic systems can be operated either in static or in dynamic mode. The example described above for the co-cultivation of cancer and immune cells was operated in a static mode in order to allow undisturbed migration of immune cells through the gap towards the cancer cells and to avoid effects of flow-induced sheer stress. Static mode is also often used during cell loading and cell adhesion in separate culture chambers. In contrast, other applications necessitate dynamic mode enabled by micropumps. This allows precise control of the cellular environment by ensuring a continuous supply of fresh media as well as a removal of metabolites. Moreover, the applied pressure and resulting sheer stress may also result in the formation of cell morphology closer to *in vivo* conditions. Within the context of the cellular systems summarized in this review article, dynamic mode microfluidics offers several key advantages: It allows for the development of highly miniaturized and parallelized dynamic cultivation platforms including reservoirs and channels for medium and supplement supply, as well as bypasses and outlets for the integration of analytics. Thereby, the consumption of samples, media, and supplements is minimized, and blood flow can be mimicked.

Moreover, the dimensions of microfluidic systems are close to the scales of biological systems. This allows the simulation of the cellular microenvironment including, e.g., diffusion barriers and/or adsorption of substances and resulting concentration profiles. Besides, the major advantage of the microfluidic cell culture system is the better reflection of the interaction of different cell types or organs. In this scenario, one has to consider that, e.g., the administration of a drug does not result in an even distribution of the drug in all cell types and organs. In particular, the concentration will be highest at the point of administration while, in distinct regions of the body, the drug will occur in a diluted concentration. These differences in drug distribution will be further increased by adsorption of the drug within certain tissues resulting in lower bioavailability. Moreover, tissues do not only adsorb drugs in a passive way, cells also metabolize the drug. Therefore, peripheral cells might hardly see any active drug but rather be challenged by metabolites, which might seriously contribute to toxic effects.

In summary, microfluidics allow for the design of co-culture chips helpful for the simulation of cell–cell interactions [178]. As an extension of these systems, OOC systems have been developed for a better understanding and simulation of complex networks present in the human body.

#### 2.4.2. Organ-on-a-Chip Systems

The US Food and Drug Administration (FDA) reported in 2004 that 92% of new potential drugs fail their approval in first clinical trials, although they pass successfully all *in vitro* and *in vivo* preclinical experiments [182]. At this point of development, the drug is applied to healthy people. Here, serious drawbacks of current drug screening systems become obvious: While conventional *in vitro* studies using human cells fail to imitate complex cellular networks, results of *in vivo* animal studies are not readily transferable to humans based on differences in physiology and metabolism.

To improve the drug safety system and increase the efficiency of the drug screening process, as well as to reduce animal use in *in vivo* studies, novel well-designed human test systems are needed. In this context, OOC systems arose as a platform combining the advantages of TMA (namely the involvement of different cell types representing a functional unit of the tissue) and CMA (especially the application of living cells comprising metabolic activity), in combination with the benefits of microfluidic systems elaborated above.

In the simplest case, the OOC aims to simulate one specific organ [183]. For example, microfluidic systems with integrated membranes have been used to simulate the human gut. In this case, the membrane was used as a matrix for human intestine epithelial cell adhesion. By applying fluid flow, sheer stress was introduced and cyclic strain was used to mimic peristaltic motion. Under these conditions, columnar epithelium developed and folded into structures recapitulating the structure of intestinal villi. This system was used to investigate the symbiotic relationship between epithelial cells and *Lactobacillus rhamnosus* as a commensal intestinal microbe [184]. Co-cultivation of epithelial cells with bacteria improved the barrier function as already observed for the human gut *in vivo*. Thus, the gut-on-a-chip was supposed to be a valuable tool for drug screening tests.

By cultivation of renal cells, kidney-on-a-chip systems have been developed. For kidney cells, fluid shear stress has shown to be an important factor: Under physiological conditions, the cells are subjected to luminal fluid shear stress, which is a key modulator for cellular signal transduction [185], the organization of the cytoskeleton, and the formation of cellular junctions. By simulation of this fluid flow, kidney models with more realistic physiology can be constructed in a microfluidic device. Another organ already transferred to a microfluidic chip is the lung. By recapitulation of the complex microstructure of the tissue, and simulation of the mechanical stress induced by breathing, different lung-on-a-chip devices have been built and used for drug screening, as reviewed by Doryab *et al.* [186].

While these single-OOC devices have already been successfully applied to drug screening procedures and have been helpful towards a better understanding of diseases, the ultimate vision is a complete human-on-a-chip device. As a step forward to this direction, multi-organ-on-a-chip (multi-OOC) systems have attracted attention in recent years [187]. One major application of multi-OOC systems is pharmacokinetic drug toxicity screening. The action of a drug in the human body is governed by the complex interaction of several processes including the absorption of the drug by cells, its distribution in different cells, tissues, and organs, its metabolization within the different cells and organs, and its elimination (ADME). Moreover, *in vivo* microenvironments are often highly dynamic and heterogeneous with respect of blood flow, accounting for convective drug transport and diffusion barriers resulting in the formation of concentration profiles. These processes cannot be mimicked by static CMAs and single-OOC systems but can be modeled in multi-OOC systems.

By the combination of different types of living tissues, and their interconnection by microfluidics, multi-OOCs are able to mimic *in vivo* drug response [188]. For example, Maschmeyer *et al.* [189] have developed a four-OOC system for the co-culture of human intestines, livers, skin, and kidneys. Therefore, human skin biopsies and 3D-villi-like structured intestine models have been integrated into the microfluidic systems. Liver was simulated by 3D spheroids and kidney by a monolayer of human proximal tubule epithelial cells on a membrane. Fluid excreted by the kidney cells was removed via the membrane. Using a peristaltic micropump, pulsatile media flow was applied to the channels interconnecting the four tissues. By the choice of integrated tissues, the authors were able to simulate several key parameters for *in vivo* drug fate: Through the kidney cells, drug excretion can be simulated, while the liver cells contribute a model for first path metabolism. The intestine compartment may act as a simulation unit for oral administration of the drug. The chip maintained homeostasis for at least 28 days and was therefore suggested as a device for ADME profiling and repeated-dose systemic toxicity testing of drug candidates [189].

This example demonstrates the power and potential of state-of-the-art multi-OOC devices. In the future, these efforts might result in a complete human-on-a-chip system allowing a mimicking of whole body responses. Such systems might not only be useful in comprehensive drug screening and toxicity assays, but may also help to gain better insights into the mechanisms of various diseases [190].

## 3. Current Limitations

The advantage of microarray technology is the very high specificity of each recognition event. Contrary to molecular identification, the analysis of cells is less specific, a fact mainly caused by cellular complexity. This disadvantage is balanced out by the main strength of using living systems: online and/or real-time investigation of biological effects. Despite this promising advantage, most investigations are unfortunately only proof-of-principle studies without further applications. Often, the complexity and scale of throughput are limited by the analytical technique, data acquisition, and data evaluation. These are own separate investigation parts within the process of microarray establishment. Moreover, high-throughput read out, e.g., with automated microscopes, automated segmentation, and analytic algorithms have to be adapted for the analysis of multiple signals. In particular, long-term studies of activity, viability, or stimulation of cells on LCMAs require these complex, well-matched techniques.

One big challenge is the usage of more than one cell type on the same microarray. Often each cell type needs its individual cultivation condition. Cross-contaminations of different cell types through cell migration have to be avoided using physical or chemical borders for an adequate identification of the cell positions. Berthuy *et al.* [73] mainly discussed, in their recently published review, strategies for the preparation of multiplexed LCMAs. Additionally, the cross-talk between the cells is an important limitation. Cells influence each other via the secretion of cytokines or several other factors, which are taken up by other cells. If different cell types are not separated from each other, their biochemical signals may change cell responses and affect study results. This applies not only to cells of different tissue origin, but also to cells that are transfected with different genes. Assays analyzing the supernatant are not applicable in this case. Although this is a desired effect in co-cultivation studies, it has to be considered in all investigations, where different cells are cultivated next to each other.

Commercially available DNA-microarrays are ready-to-use and storable; TMAs and CMAs of fixed cells are stable for several months. Even microarrays printed with plasmids or other nucleotides can be stored and used for transfected cell microarrays in another laboratory. In contrast, microarrays containing living cells have to be freshly prepared each time before an investigation can take place. Under consideration of this aspect, this type of microarray is more time-consuming and complex. The laboratories, which work with LCMAs, have to have a cell culture lab and adequate analytics matching their application, as well as access to microarray preparation techniques, such as lithography or nanoprinter devices. Contrary to this, laboratories applying samples onto TMAs and fixed CMAs do not need any kind of microarray preparation technique, such as tissue/cell isolation, hollow needles, and donor/acceptor blocks or a microtome. Instead, TMAs and CMAs can be commercially obtained. If LCMAs could be prepared and stored in one place and later used for investigations at a different laboratory, this would be extremely helpful and time-saving. Laboratories would also be independent from microarray preparation techniques and could thus be more focused on developing applications.

## 4. Conclusions and Future Directions

New fields of application of microarray technology have been developed for use in medicine, biology, and toxicology. The fundamentals of microarray technology have been used to design new special techniques. Microarrays are no longer only based on oligonucleotides, antibodies, proteins, or aptamers. Even cells (fixed or alive) can be immobilized on slides creating special cell microarrays. Current cell arrays can be categorized into tissue, cell lines, primary cells, and organ biopsies. Furthermore, cellular microarrays can be classified depending on diverse criteria such as fixed tissue or systemic approaches, static or dynamic cultivation conditions, and 2D or 3D cell culture. Predominantly, living cell microarrays are currently used to analyze cellular behavior and for pharmaceutical drug testing. In this article, we focused on self-spotted glass slide living cell arrays, which are a popular format. The glass slides are coated with functional groups enabling the attachment of cells to the glass surface. After attachment, cells proliferate on the glass surface comparable to traditional plastic cultivation flasks. These microarrays can be stored under defined conditions. In the case of human tissues, which are rare and hard to obtain, strategies to enrich well characterized cells are important. LCMAs can be used to overcome these limitations. This approach is enabling minimal invasive analysis of patients and will be useful for biomarker detection, single-cell analysis, personalized medicine, and point-of-care diagnostic. Important applications of LCMAs include the discrimination between different tissue phenotypes and the discovery of previously unknown tissue subtypes, e.g., in cancer research. Usually, studies are performed with hundreds of thousands or even millions of cells. In some cases, it is not possible to collect large numbers of cells, which makes their analysis (e.g., transcriptome) almost impossible. LCMAs provide an alternative opportunity to work with only a few cells. To improve the detection and treatment of diseases and to analyze fundamental biological principles, new approaches to single-cell analysis must be developed.

To create 3D cell cultures, drops of hydrogels, synthetic scaffolds, or cell organoids can be attached to the microarray platform. Being more physiologically relevant, these 3D systems possess challenges in analytics and image acquisition. However, 3D cell microarrays have great perspectives in personalized drug screening in the improvement of preclinical testing (e.g., for anti-cancer drugs and substances, which help to restore cell specific cell functions).

With the help of this new method, a high-throughput screening for toxicology, protein expression and production, micro RNA expression, drug testing, or the stimulus-response relationships of living cells is possible. To acquire not only results of toxicology, but also a better understanding of stimulus-response assays, a suitable monitoring of living cells is essential. For example, a very common method to monitor a stimulus-response relationship is the use of impedance measurements (ECIS). Here, a change of the cell shape caused by a drug or chemical or any other kind of stimulus can be measured easily. This technique can show much more sensitive results of a dose-response than any kind of colorimetric method. Simple cell cultures or cell lines can be used in early screens and for more complex questions; e.g., organoid cultures are an adequate screening system.

Finally, there is a clear value of living cell microarrays to enable the analysis of different cell types and treatment conditions on a miniaturized high-throughput platform, thereby minimizing costs, reagents, drugs, and experimental variance due to very small sample volumes.

## Abbreviations

2D	Two-dimensional
3D	Three-dimensional
ADME	Absorption, distribution, metabolism, and excretion
APTES	(3-Aminopropyl)triethoxysilane
CD	Cluster of Differentiation
CHO	Chinese Hamster Ovary
CMA	Cell microarray
CSC	Cancer stem cell
CTC	Circulating tumor cells
DIC	Differential interference contrast
DNA	Deoxyribonucleic acid
ECIS	Electric cell-substrate impedance sensor
ECM	Extracellular matrix
EDC	1-Ethyl-3-(3-dimethylaminopropyl)carbodiimide
EpCAM	Epithelial Cell Adhesion Molecule
ESC	Embryonic stem cell
FA-Mont	Folic acid modified montmorillonite clay
FDA	Food and Drug Administration
GFP	Green fluorescent protein
IgG	Immunoglobulin G
iPSC	Induced pluripotent stem cell
LCMA	Living cell microarray
MEA	Multi-electrode array
MEMS	Micro-electromechanical systems
MRI	Magnetic resonance imaging
mRNA	Messenger RNA
MSC	Mesenchymal stem cell
NHS	*N*‑Hydroxysuccinimide
OOC	Organ-on-a-chip
PCR	Polymerase chain reaction
PEG	Polyethylene glycol
PLA	Polylactic acid
PLL	Poly-l-lysine
PSMA	Prostate-specific membrane antigen
RNA	Ribonucleic acid
RNAi	RNA interference
SC	Stem cell
shRNA	Short hairpin RNA
siRNA	Small interfering RNA
SPR	Surface plasmon resonance
TMA	Tissue microarray
UV	Ultraviolet
